# Muscle Regeneration and Function in Sports: A Focus on Vitamin D

**DOI:** 10.3390/medicina57101015

**Published:** 2021-09-25

**Authors:** Giovanni Iolascon, Antimo Moretti, Marco Paoletta, Sara Liguori, Ombretta Di Munno

**Affiliations:** 1 Department of Medical and Surgical Specialties and Dentistry, University of Campania “Luigi Vanvitelli”, Via de Crecchio, 6, 80138 Naples, Italy; giovanni.iolascon@unicampania.it (G.I.); marco.paoletta@unicampania.it (M.P.); sara.liguori@unicampania.it (S.L.); 2 Rheumatology Unit, Department of Clinical and Experimental Medicine, University of Pisa, 56122 Pisa, Italy; ombretta.dimunno@med.unipi.it

**Keywords:** vitamin D, satellite cells, skeletal muscle, muscle fibers, skeletal, return to sport, sports, athletes

## Abstract

Muscle is one of the main targets for the biological effects of vitamin D. This hormone modulates several functions of skeletal muscles, from development to tissue repair after injury, through genomic and non-genomic mechanisms. Vitamin D deficiency and supplementation seem to significantly affect muscle strength in different populations, including athletes, although optimal serum 25(OH)D3 level for sport performance has not been defined so far. Additionally, vitamin D deficiency results in myopathy characterized by fast-twitch fiber atrophy, fatty infiltration, and fibrosis. However, less is known about regenerative effects of vitamin D supplementation after sport-related muscle injuries. Vitamin D receptor (VDR) is particularly expressed in the embryonic mesoderm during intrauterine life and in satellite cells at all stages of life for recovery of the skeletal muscle after injury. Vitamin D supplementation enhances muscle differentiation, growth, and regeneration by increasing the expression of myogenic factors in satellite cells. The objective of this narrative review is to describe the role of vitamin D in sport-related muscle injury and tissue regeneration.

## 1. Introduction

Since its identification as an effective cure for nutritional rickets, much of the scientific research on the biology of vitamin D is directed at its role in controlling the homeostatic mechanisms of calcium. The discovery that the vitamin D receptor (VDR) is widely expressed in many tissues has prompted a series of studies aimed at understanding its roles in physiological responses unrelated to calcium homeostasis. In the last few decades, huge attention was paid to the biological functions of this secosteroid on skeletal muscle physiopathology, from contraction to tissue regeneration after immobilization or injury [1,2]. The physiological effects of vitamin D, in muscle as in other tissues, involve genomic and non-genomic mechanisms that are related to each other in crosstalk in the intracellular signaling mechanisms of vitamin D [3,4]. Thousands of vitamin D-responsive genes were identified (e.g., calbindin-D9K, osteocalcin, osteopontin), many of which are key players in skeletal muscle health being involved in protein synthesis and muscle performance [5]. Experimental data showed that serum 25(OH)D3 and vitamin D supplementation significantly affect muscle strength in several populations, such as older and younger people [6,7,8,9,10,11,12]. Indeed, vitamin D receptor (VDR) is widely expressed on the skeletal muscle cells, with potential effects on the de novo protein synthesis, thus contributing to the muscle hypertrophy [13]. Moreover, among the non-genomic effects of VDR, an improvement of muscle contraction was observed, due to the modification of intracellular calcium fluxes and enhancement of the interaction between myosin and actin in the sarcomere, a crucial event for muscle function [14].

Vitamin D deficiency was also observed among athletes, not only in those practicing indoor activities (e.g., basketball and dance) [15], but also in football and soccer players [16]. In athletes, vitamin D deficiency may be attributable not only to training settings (indoor vs. outdoor), but also to timing of training (early- or late-day), geographic location, skin pigmentation, and sunscreen use [16].

In this context, a key issue is the definition of both vitamin D status (i.e., serum 25(OH)D3.) and optimal levels, particularly among the athletes.

In the general population, concentrations of >30 ng/mL are considered acceptable in terms of benefits on bone health. On the other hand, the specific threshold for reaching optimal sport performance is not established in athletes [17]; although, vitamin D supplementation is required for values below 12 ng/mL [14,18].

Historically, it was well known that vitamin D deficiency is characterized by myopathy (i.e., rickets-associated myopathy) with muscle fiber atrophy accompanied by fatty infiltration and fibrosis, particularly affecting fast-twitch muscle fibers (i.e., type II), with consequent slow peak muscle contraction [19]. Moreover, immunomodulatory effects of vitamin D might be also useful to counteract increased pro-inflammatory cytokines (e.g., TNF-α) after intense exercise [20].

Although evidence suggests multiple benefits of vitamin D supplementation for skeletal muscle to improve athletic performance [16], less is known about the muscle regenerative effects of this hormone after sport-related injuries. Athletes are the individuals most exposed to muscle damage, considering its occurrence after bruising or because of high-intensity resistance training, particularly eccentric exercise [21,22]. Therefore, this narrative review aims to elucidate the role of vitamin D in the pathophysiology of muscle injury and tissue regeneration with a focus on sport.

## 2. Vitamin D and Skeletal Muscle: Basic Concepts

The striated muscle represents about 40% of the total body mass and is critical for the posture and global and selective body movements [23]. Muscle fiber is the basic functional unit of striated muscle and is composed of syncytium of multinucleated cells. Muscle fibers have different properties and are classified according to their metabolic activity and the consequent contraction type and speed. Type I fibers are characterized by an oxidative metabolism that produce energy to support a protracted and fatigue-resistant contraction. Fast twitch (type II) fibers can be glycolytic and oxidative (fast-twitch oxidative, FOG) or glycolytic only (fast-twitch glycolytic, FG) and are involved in faster contraction [24,25]. Striated muscles contain different percentages of fiber type which determine huge differences in contraction speed and muscle strength [26,27]. Furthermore, the functional activity of the muscle fibers changes over time since they can change in size and even convert from one type to another to adapt to different functional requirements [28]. Muscle responds to various biochemical stimuli. Due to its characteristics as an organ that produces and responds to various substances present in the blood circulation, the muscle is now identified as an endocrine organ [29]. In fact, it modifies its structural and functional features in response, for example, to changes in the serum levels of insulin, GH (growth hormone), glucocorticoids, thyroid hormones, and vitamin D [1,30,31,32,33]. In turn, the muscle produces some substances with endocrine activity (i.e., myokines) which have systemic effects modulating glucose uptake, fat oxidation, gluconeogenesis, bone mass and fracture healing [34]. Among several hormones affecting striated muscle, vitamin D influences its structure and function during the different stages of life, starting from embryonic development up to ageing, and it is involved also in the mechanisms of muscle repair after trauma [2,7,8,9,10].

Vitamin D receptor is localized both in the cytoplasm and in the nucleus of muscle fibers, to mediate non-genomic and genomic actions [35,36]. Moreover, the amount of VDR is significantly higher in immature myoblasts and muscle cell precursors than in differentiated myotubes or mature muscle fiber [37]. It should be emphasized that in adult animals that suffered muscle injuries, or that were subjected to stressful physical exercise, there was a significant upregulation of the VDR on the muscle fibers being repaired, confirming a prominent role of vitamin D both on cellular precursors and on muscle fibers during tissue regeneration [38]. Vitamin D acts on muscle tissue mainly through two mechanisms, by activating specific areas of the genome (genomic pathway) with consequent structural and functional plastic modifications of the muscle in the long-term, and by a faster mechanism (non-genomic pathway) that affects muscle contraction through the modification of intracellular calcium fluxes [3]. In more detail, through the genomic pathway, vitamin D stimulates the proliferation and differentiation of muscle cells by modulating gene transcription in myoblasts, resulting in an increase in the synthesis of specific muscle proteins, such as myosin and calcium-binding protein. 1α,25(OH)2D3 enhances muscle differentiation, growth, and regeneration by increasing the expression of several myogenic factors (i.e., MYOD, MYOG, MYH1, and MYC type II, muscle troponin I and T) in satellite cells as well as of other key factors involved in muscle growth and regeneration (i.e., IGF, FGF, and TGF-β) [39].

In addition to modulating calcium absorption, vitamin D participates in the metabolism of phosphate for cellular energy needs [40]. Through the non-genomic pathway, vitamin D regulates the calcium-mediated action of second messengers to enhance muscle contraction. The 1,25-(OH)2D3 acts on the SOC/TRPC3 dependent voltage channels to regulate the intracellular levels of calcium and, therefore, the excitation–contraction coupling [3]. The beneficial effects of vitamin D on the skeletal muscle depend also on other mechanisms that significantly affect muscle microarchitecture and physical performance. This hormone inhibits the differentiation of myogenic precursors into adipocytes thus limiting the accumulation of intra- and intermuscular fat [41]. Moreover, vitamin D modulates the main negative regulator of muscle growth, the myostatin [42]. In particular, 1α,25-(OH)2D3 increased the expression of follistatin (FST), an inhibitor of myostatin, and reduced the expression of myostatin in satellite cells [39].

## 3. Vitamin D and Sport-Related Muscle Injury: From Biology to Clinical Practice

The role of vitamin D in muscle development is clearly suggested by the early presence of VDR in the embryonic mesoderm and in muscle precursor cells (i.e., satellite cells) at all stages of life [43]. It is evident that this hormone must necessarily play a leading role both in the formation and growth of the muscular system during intrauterine life and in the clinical recovery of the skeletal muscle after a trauma that has altered its structure and/or function. If we consider that the increased expression of VDR in cultured C2C12 cells (myoblasts) treated with 1α,25(OH)2D3 and 25(OH)D3 alters their proliferation and differentiation [44], it might be hypothesized that vitamin D deficiency can cause an impairment in the healing process in patients who have suffered a direct trauma or have sustained a damaging functional stress (e.g., eccentric exercise). The expressions of VDR and 25-hydroxyvitamin D-1α-hydroxylase (CYP27B1) were demonstrated in myoblasts and myotubes [45]. VDR is localized in nuclei of myoblasts, and in cytoplasm of myotubes, probably because of the increased expression of RXR in myotubes that is associated with increased cytoplasmic localization of VDR [46]. However, increased nuclear VDR was documented in myotubes treated with calcitriol. 25(OH)D3 inhibited myoblasts proliferation and increased VDR expression, suggesting the conversion of 25(OH)D3 to 1α,25(OH)2D3. However, the growth-suppressive effect of 25(OH)D3 on the myoblasts was dropped after the inactivation of the CYP27B1, suggesting a key role of 1α- hydroxylase in modulating the proliferation of muscle cells by 25(OH)D3. Moreover, VDR and CYP27B1 were significantly upregulated in myonuclei and cytoplasm, respectively, of the regenerating muscle fibers [45].

Even negligible muscle injuries, such as acute microinjury during resistance training, increase VDR and CYP27B1 suggesting the enhanced sensitivity of injured and healing muscle to vitamin D [47]. Morphological changes occur in muscle fibers after acute trauma or during eccentric contraction, such as myofiber necrosis and inflammatory responses that result in the widening of perimysium among fascicles, and the separation of myofibers within the same fascicles [48]. Muscle regeneration is a complex process based on the activation, differentiation, and formation of new myofibers from muscle-specific stem cells (i.e., satellite cells) under the control of the myogenic and mitogenic regulatory factors [49]. The satellite cells are located between the muscle fibers and the basal membrane, and they can be activated when necessary, making the muscle able to heal after tissue damage. After trauma, satellite cells are activated and guide muscle repair. Their activation and differentiation are regulated by complex paracrine signals from neighboring cells (e.g., fibroblasts, endothelial cells, and macrophages), intracellular signals (e.g., Wnt and Notch) [50,51], myogenic regulatory factors (e.g., myf5 and myoD) [52]. After activation, the satellite cells can replicate and some of them differentiate into myoblasts, based on the expression of Pax7 compared to myf5 [49]. Activated satellite cells differentiate into myoblasts and myocytes that express specific markers (i.e., (Pax7+, Myf5+, and MyoD+ in myoblasts, and Pax7−, MyoD+, myogenin+, and MRF4+ in myocytes) [53]. Finally, fused myocytes form multinucleated myotubes that mature into myofibers. To specifically address muscle repair after exercise-related tissue injuries, Hyldahl et al. investigated satellite cell activity and inflammatory milieu in human skeletal muscle following eccentric (ECC) or concentric contractions (CON). They found that only ECC induced high levels of Xin (i.e., striated muscle-specific protein) in the sarcolemma, and inflammatory cytokines, such as interferon gamma-induced protein 10 (IP-10) and monocyte chemotactic protein 1 (MCP-1). Moreover, ECC-induced muscle damage significantly increased satellite cell proliferation (+27%) compared to CON in the early phase of injury (24 hrs) [54]. After muscle damage, several growth factors are released activating nuclear pathways in quiescent satellite cells [55].

Vitamin D modulates muscle repair by regulating satellite cell proliferation and differentiation as well as mitochondrial density and function [56]. VDR and Pax7 are colocalized in the nuclei of satellite cells. After muscle injury, overexpression of both VDR and Pax7 occurs, resulting in inhibition of proliferation and stimulating differentiation of satellite cells [46,57,58]. Increased expression of VDR after muscle injury also positively affects mitochondrial health by increasing biogenesis and reducing oxidative stress. Moreover, administration of vitamin D inhibits proliferation and increases differentiation of myoblasts as well as oxygen consumption in mitochondria, that might be the fuel for myotube formation [59]. Whereas vitamin D deficiency adversely affects mitochondrial function by reducing adenosine triphosphate (ATP) production and increasing oxidative stress, as demonstrated in VDR-knockout myoblasts [60], thus hindering muscle regeneration. Mitochondrial function is critical for muscle repair after injury considering its role in modulating satellite cells activity [61], as demonstrated by reduced oxidative capacity in quiescent satellite cells [62]. Moreover, modulation of mitochondrial mitophagy seems to be necessary to satellite cell function, as demonstrated in mouse models knock-out for Parkin, an E3 ubiquitin ligase [63], that showed reduced differentiation of satellite cells and, consequently, poor muscle regeneration. These data confirm that mitophagy favors the increase and maintenance of the satellite cells pool.

Traditionally, the role of vitamin D in calcium metabolism was widely investigated, whereas the effect of this hormone on phosphorus metabolism was not addressed so extensively and dates back mainly to the 1970s and 1980s, although the effects of vitamin D on skeletal muscle were attributed to the modulation of phosphate metabolism [64,65]. Phosphate is an essential substrate for ATP production and protein synthesis. The treatment of vitamin D deficient rats and chicks with 25(OH)D3 resulted in a significant increase in phosphate uptake and stimulation of ATP synthesis in muscle cells [64]. The active form of vitamin D [i.e., 1α,25(OH)2D3] seems to significantly affect mitochondrial health in muscle cells by modulating calcium metabolism. Animal (i.e., chicks) and human models of vitamin D deficiency demonstrated both impaired oxidative phosphorylation and calcium uptake in muscle mitochondria [66,67]. On the other hand, muscle cells exposed to 1α,25(OH)2D3 increased oxygen consumption and ATP production, while no effect was reported by administrating 25(OH)D3. However, these effects were not observed after the direct treatment of mitochondria with 1α,25(OH)2D3, suggesting a VDR-dependent mechanism [68].

Vitamin D supplementation also mitigates the effects of muscle damage secondary to high intensity exercise as demonstrated in both animal and human studies (on Sprague–Dawley rats and young men, respectively) [69,70]. During a muscle injury, administration of vitamin D reduces the production of stress-related proteins (p38 MAPK, ERK1/2, IKK, IĸB), inflammatory cytokines (TNF-α, IL-6) and oxidative stress [69]. Moreover, a more rapid recovery of the contractile force of the damaged muscle after vitamin D supplementation was reported [71], whereas, in an experimental model of C57BL/6 mice with an induced muscle injury, excessive doses of 1α,25(OH)D3 or its intramuscular administration did not have beneficial effects on muscle regeneration but can even negatively affect the activity of satellite cells thus compromising the formation of muscle fibers [57].

**Figure 1:**
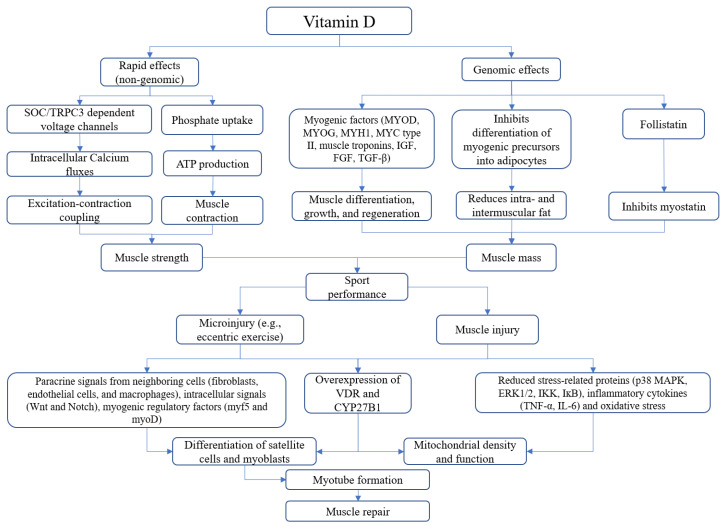
Vitamin D-mediated mechanisms affecting skeletal muscle performance and healing. Vitamin D produces non-genomic effects that affect calcium and phosphate uptake within muscle to sustain contraction, while long-term effects (genomic) of this hormone influence the release of myogenic factors and reduce the accumulation of fat tissue within muscle fibers as well as muscle catabolism. After direct or indirect muscle injury, three main events occur: paracrine signals by neighboring cells stimulate the release of myogenic mediators, overexpression of VDR and CYP27B1 and reduction of oxidative stress.

In Figure 1 is reported a summary of multiple vitamin D-mediated mechanisms affecting skeletal muscle health.

Even in humans, vitamin D status significantly affects muscle repair after injury. It was documented that the serum levels of 25(OH)D3 before exercise are inversely related to post-exercise muscle weakness in a healthy adult male population [71]. Interventional studies have reported a beneficial effect of vitamin D supplementation on muscle recovery in adult males exposed to muscle injury from repetitive eccentric contractions [71]. As is known, vitamin D deficiency contributes to muscle wasting by promoting oxidative stress, mitochondrial dysfunction, reduction of superoxide dismutase (SOD), and favoring the production of free radicals within muscle fibers [72]. Normalization of the vitamin D status in severely deficient subjects significantly improves mitochondrial function and oxidative phosphorylation of muscle fiber after exercise [66]. Furthermore, vitamin D supplementation might reduce muscle fatigue since serum levels of 25(OH)D3 are inversely correlated with those of lactic acid, creatine kinase (CK) and total antioxidant activity after exercise [73].

Another issue to be clarified is the effect of initiation timing of vitamin D treatment after injury, as suggested by experimental data. Subcutaneous administration of cholecalciferol to male Wistar rats soon after crush injury resulted in enhanced proliferation of immune cells, such as macrophages, as well as satellite cells, along with a reduction of cell apoptosis that preserved muscle structure [74]. However, delayed intramuscular administration of calcitriol decreased satellite cells differentiation and increased muscular fibrosis [57].

The serum 25(OH)D3 is a key issue to be considered in the context of vitamin D treatment. Young males with vitamin D deficiency undergoing exercise-induced muscle injury received cholecalciferol (4000 IU daily for 6 weeks) showed significant recovery of peak torque at 2- and 7-days post exercise [75]. Moreover, myoblasts from biopsies of the same population received mechanical injury and were cultured with calcitriol. This intervention improved myotube fusion and hypertrophy at 7- and 10-days after injury. These findings suggest potential benefits of treating vitamin D deficiency in athletes. Interestingly, vitamin D status seems to be involved also in the occurrence of sport-related muscle injury, as suggested by a study including National Football League (NFL) players [76]. In this cohort, vitamin D deficiency significantly increased the risk of strain at lower limb or core muscles (Odds Ratio, OR 1.86), particularly at hamstrings (OR 3.61) compared to athletes with normal serum 25(OH)D3. Similar findings were reported also in swimmers, where low serum 25(OH)D3 levels increased the risk of muscle injuries (+77%) [77]. Vitamin D supplementation reduced the risk of muscle injury also in elite ballet dancers [78]. These athletes received oral supplementation of cholecalciferol (2000 IU per day) for 4 months during winter, reporting fewer muscle injuries compared to controls that did not receive vitamin D [78]. Moreover, increasing serum 25(OH)D3 reduces muscle weakness after exercise-induced muscle injury, thus reducing recovery times [79]. Finally, the vitamin D status should not be overlooked also during periods of inactivity due to the sport-related injury. Experimental data suggest that vitamin D deficiency worsens immobilization-induced muscle wasting. In VDR-knock-out mice models, limb immobilization resulted in more severe muscle atrophy than controls. Interestingly, more pronounced muscle atrophy and increased expression of pro-inflammatory cytokines promoted by immobilization (i.e., TNF-α) were reported in mice with neural crest-specific VDR-deficiency compared to those with muscle-specific VDR-deficiency. This finding opens new scenarios on the role of the vitamin D system as a regulator of muscle mass through its action on the central nervous system [80].

## 4. Conclusions

Vitamin D has documented effects on muscle regeneration through mechanisms and biological pathways that mainly depend on the interaction with the pool of satellite cells within muscle and that are particularly active during recovery from a traumatic event to enhance the structural and functional restoration of the muscle. However, the number of studies addressing the role of vitamin D on muscle repair in athletes is relatively small and well-designed randomized controlled trials are lacking. Therefore, future research should be directed to improve knowledge about the clinical benefits of vitamin D supplementation in sport-related muscle injury.
